# Permeability and Disintegration Characteristics of Loess Solidified by Guar Gum and Basalt Fiber

**DOI:** 10.3390/ma17133150

**Published:** 2024-06-27

**Authors:** Yu Xi, Mingming Sun, Huanhuan Li, Gang Li, Pengzhou Wang, Li Li

**Affiliations:** 1 Shaanxi Key Laboratory of Safety and Durability of Concrete Structures, Xijing University, Xi’an 710123, China; 2 School of Civil Engineering and Architecture, NingboTech University, Ningbo 315100, China; 3 China Construction Fourth Engineering Division Corp., Ltd., Guangzhou 511400, China

**Keywords:** guar gum, basalt fiber, loess, microscopic structure, solidified mechanism

## Abstract

Loess has the characteristics of loose, large pore ratio, and strong water sensitivity. Once it encounters water, its structure is damaged easily and its strength is degraded, causing a degree of subgrade settlement. The water sensitivity of loess can be evaluated by permeability and disintegration tests. This study analyzes the effects of guar gum content, basalt fiber content, and basalt fiber length on the permeability and disintegration characteristics of solidified loess. The microstructure of loess was studied through scanning electron microscopy (SEM) testing, revealing the synergistic solidification mechanism of guar gum and basalt fibers. A permeability model was established through regression analysis with guar gum content, confining pressure, basalt fiber content, and length. The research results indicate that the addition of guar gum reduces the permeability of solidified loess, the addition of fiber improves the overall strength, and the addition of guar gum and basalt fiber improves the disintegration resistance. When the guar gum content is 1.00%, the permeability coefficient and disintegration rate of solidified soil are reduced by 50.50% and 94.10%, respectively. When the guar gum content is 1.00%, the basalt fiber length is 12 mm, and the fiber content is 1.00%, the permeability of the solidified soil decreases by 31.9%, and the disintegration rate is 4.80%. The permeability model has a good fitting effect and is suitable for predicting the permeability of loess reinforced with guar gum and basalt fiber composite. This research is of vital theoretical worth and great scientific significance for guidelines on practicing loess solidification engineering.

## 1. Introduction

Loess is an aeolian sediment and is composed of silt particles [1,2,3], which causes the structure to be relatively loose and easily eroded by water or wind. When the loess is subjected to wind and water erosion, the safety stability coefficient *K* is less than 1, indicating a very low level of stability. When it is wet, its structure is damaged easily and rapidly, its strength is degraded, its total volume is reduced or collapsed, and additional sedimentation occurs, causing a degree of subgrade settlement [4,5,6]. With the implementation of policies such as the Belt and Road Initiative, the Silk Road, and the 14th Five-Year Plan, more and more infrastructure is being built in the loess regions, causing a large number of loess engineering and geological problems. If not handled properly, these problems will affect the safety and stability of construction projects [7,8]. Some scholars have discovered that guar gum or xanthan gum can effectively solidify soil. Bagheri et al. [9] pointed out that untreated soil completely disintegrated after four hours of immersion, but the xanthan-solidified soil remained intact after two days of immersion, indicating that xanthan gum can effectively reduce the water sensitivity of soil. Hamza et al. [10] declared that guar gum forms hydrogel when it meets water and has a covering effect on soil particles, which not only fills the soil pores and reduces the permeability but also improves the compactness. Jia et al. [11] pointed out that guar gum can effectively improve the mechanical behaviors of loess in the process of shearing and disintegration; with an increase in guar gum content, the disintegration rate and the permeability coefficient decrease. Han et al. [12] pointed out that the initial protective effect of guar gum mixed soil is better than that of polypropylene fiber-reinforced soil.

In recent years, fiber-reinforced polymers have gradually become research hotspots, and many scholars have applied them to civil engineering [13]. Zhang et al. [14] declared that fibers can improve the water stability of cementitious soil and delay the disintegration rate. Yuan et al. [15] found that when polypropylene fibers were added to lime loess, their permeability was improved, indicating that polypropylene fibers can create seepage channels in soil and improve soil permeability. Kannan and Sujatha. [16] declared that banana fiber can improve soil drainage characteristics. There are also studies using active agents or industrial waste to improve loess. Bai et al. [17] found that lignin calcium sulfate improves the collapse of loess, fills soil pores, connects soil particles, and reduces the water sensitivity of loess. Hou et al. [18] adopted nanomaterials and industrial waste as stabilizers for loess, which effectively improves its water stability through filling, bonding, and wrapping. Li et al. [19] found that calcium ligne-sulfonic acid could reduce the interfacial crystal spacing of minerals and the thickness of electric double layers of particles by hydrolyzing clay minerals in loess, thus improving the water stability and disintegration resistance of loess.

Most scholars have studied the mechanical properties of fiber-reinforced loess and concluded that fiber can improve the strength of soil. There are also studies indicating that guar gum or xanthan gum plays an important role in soil stability [20,21,22,23], but the study of guar gum combined with basalt fiber-solidified loess is still relatively few [24,25,26,27]. Based on previous research results, the guar gum content and fiber content were determined [11,28]. The effects of guar gum content, fiber length, and fiber content on the solidification of loess with guar gum and basalt fibers were analyzed using permeability and disintegration tests, and the optimal solidification conditions were determined by the lowest disintegration rate. The microstructure of loess was studied using a scanning electron microscopy (SEM) test, and the synergistic solidification mechanism of guar gum and basalt fiber was revealed. Based on the test results, a permeability model was established that considers the effect of guar gum content, fiber content, fiber length, and confining pressure. The research is of vital theoretical worth and great scientific significance for guidelines on loess solidification engineering.

## 2. Materials and Methods

### 2.1. Test Materials

**Figure 1:**
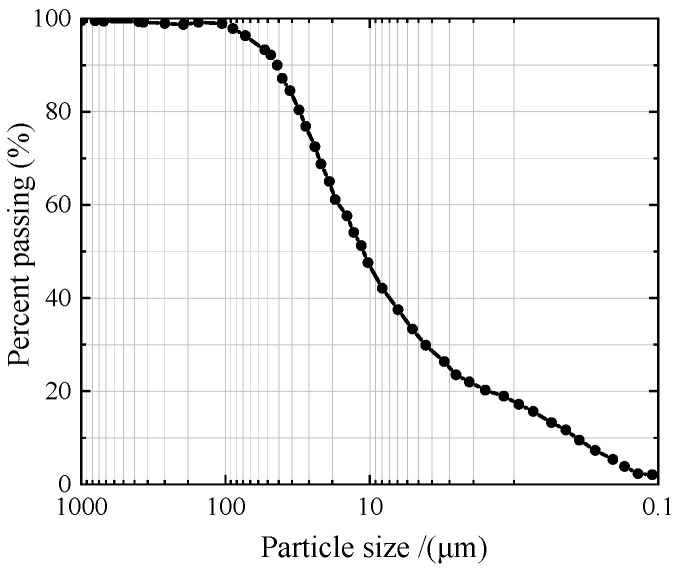
Grading curve of loess.

Loess in Western China has obvious characteristics such as collapsibility and large pores. The trial loess was gathered from a construction site in Chang’an District, Xi’an City, Shaanxi Province, and the sampling depth was between 2 m and 3 m. During the sampling process, areas with fewer plant roots are excavated. Then, sealed plastic wrap is used to store and reduce soil disturbance during transportation, avoiding direct sunlight, and the samples are refrigerated at 4 °C in a refrigerator. The grading curve of loess is shown in Figure 1. The physics ingredient indicators of the loess are listed in Table 1.

The guar gum used in the test was purchased from Henan Wonbond Chemical Co., Ltd. (Zhengzhou, China). It has a white appearance and can form viscous colloids in cold water. It is often used to thicken, solidify, and enhance the texture and stability of food. Basalt fiber is a multi-functional fiber material in bronze color. After decomposition, it is fluffy and can sink into water. The physical and mechanical properties of the property indicator of basalt fiber are shown in Table 2.

### 2.2. Sample Preparation

**Figure 2:**
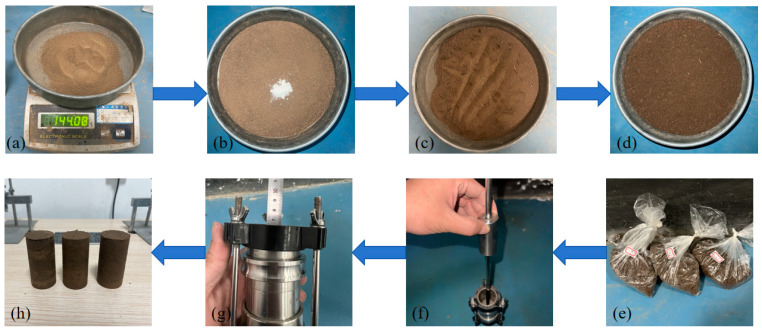
Sample preparation flow chart: (**a**) weight soil; (**b**) add guar gum and stir it evenly; (**c**) add fiber and stir it evenly; (**d**) add water and stir it evenly; (**e**) seal it and let stand; (**f**) load material and prepare sample; (**g**) control the height; (**h**) remove the mold.

According to the geotechnical testing standard (GB/T50123-2019) [29], the KTL-LDF 50 soil static triaxial testing machine was selected, and the test confining pressure was determined to be 25 kPa, 50 kPa, and 100 kPa by sampling depth. The sample size was 39.1 mm × 80 mm (diameter × height). Before sample preparation, silicon oil was evenly applied to the inner wall of the sample kit to prevent the soil material from sticking to the sample kit. The sample was prepared in five layers. To ensure compact adhesion between layers, each layer was roughened after being compacted. The sample preparation process is shown in Figure 2. The sample was prepared according to the following steps: (a) weigh soil; (b) add guar gum and stir it evenly; (c) add fiber and stir it evenly; (d) add water and stir it evenly; (e) seal it and let it stand; (f) load material and prepare sample; (g) control the height; (h) remove the mold.

### 2.3. Test Method

Tests were carried out using the KTL-LDF 50 soil static triaxial testing machine and SHY-1 disintegration tester to research the disintegration and permeability characteristics of the loess solidified by guar gum and basalt fiber. The permeability test was performed under the permeability of a constant head. The diameter of the sample is 39.1 mm, and the sample level is 80 mm. Based on the relevant research results [11,28], the content of guar gum was determined to be 0.50%, 0.75%, and 1.00%, respectively; the fiber length was determined to be 4 mm, 8 mm, and 12 mm, respectively; and the fiber content was determined to be 0.20%, 0.60%, and 1.00%, respectively. The test confining pressure was determined to be 25 kPa, 50 kPa, and 100 kPa by sampling depth. The compaction test shows that the maximum dry density of loess is 1.50 g/cm^3^, and the optimal water content is 20%. The permeability pressure was achieved by dividing the difference between base pressure and back pressure and setting it at 20 kPa. See Table 3 for the permeability test scheme. The size of the disintegration test specimen is 50 mm in diameter and 50 mm in height, and the trial scheme is shown in Table 4. The disintegration rate during the test is calculated as per Formula (1).

(1) $$D_{t}=\frac{R_{t}-R_{0}}{R_{0}}\times 100\%$$

where, *D*_t_ is the sample disintegration rate at the moment t, %; *R*_t_ is the scale indication of the floating cylinder at the same level with the water, cm; *R*_0_ is the transient stable scale indication of the floating cylinder at the same level with the water at the beginning of the test.

## 3. Results and Analysis

### 3.1. Analysis of Permeability and Disintegration Characteristics of the Loess Solidified by Guar Gum

**Figure 3:**
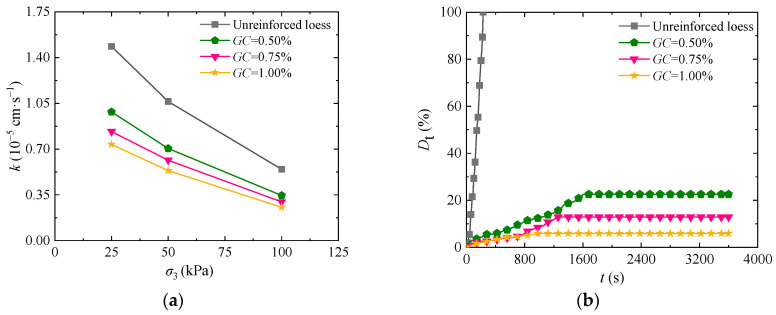
Impact of guar gum content on the loess permeability coefficients and disintegration rate: (**a**) permeability characteristics; (**b**) disintegrating characteristics.

Compared to lignin calcium sulfate, guar gum is a non-polluting green material with a short curing time [19]. To analyze the impact of guar gum on the permeability and disintegration characteristics of loess, Figure 3a presents the curve of permeability coefficient change with the guar gum content in solidified loess at different levels of confining pressure. As shown in the figure, the permeability of the loess solidified by guar gum reduces with increasing confining pressure. At the same confining pressure, the permeability of solidified loess decreases gradually by increasing the guar gum content. Concerning the primary cause, guar gum forms a hydrogel in contact with water after being added to the soil, and the hydrogel blocks the seepage passages in the soil, thus developing a physical barrier against any flow through the sample. Figure 3b is the time–history curve of the disintegration rate of solidified loess under different guar gum content. It indicates that the disintegration resistance of the loess solidified by guar gum increases significantly with the increase in guar gum content. However, note that guar gum content has a significant effect on the short-time disintegration rate curve. When the guar gum content is 0.50%, 0.75%, and 1.00%, and the disintegration time is 300 s, the disintegration rate is 5.40%, 2.56%, and 2.60%, respectively. The disintegration rate of solidified loess steadily tends to be stable after 1600 s, 1200 s, and 800 s. The main reason lies in the fact that guar gum produces a higher degree of cementation to soil particles with the increase in guar gum content. Under such circumstances, it is more difficult to decompose the loess solidified by guar gum in water [30]. By combining the permeability characteristics with the disintegration characteristics of solidified loess, we can learn that the optimal guar gum content is 1.00%.

### 3.2. Analysis of Permeability and Disintegration Characteristics of the Loess Reinforced by Basalt Fiber

**Figure 4:**
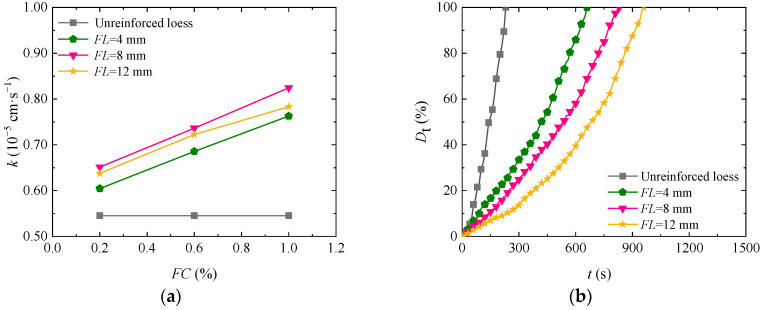
Impact of basalt fiber length on the loess permeability coefficients and disintegration rate: (**a**) permeability characteristics; (**b**) disintegrating characteristics.

Fiber length increases the seepage passage of soil mass, thus affecting the permeability and disintegration characteristics of loess. Figure 4a shows the curve of permeability coefficient change with fiber content in the loess reinforced by basalt fiber with different fiber lengths under a confining pressure of 100 kPa. The permeability of fiber-fortified loess improved with increasing fiber length and decreased after reaching a certain length. When the fiber length is constant, the permeability of reinforced loess increases slowly by increasing the fiber content. The loess has the largest permeability coefficient with an 8 mm fiber length. The main reason for the above situation is that if the fiber content is constant, the seepage passage of soil mass increases by increasing the fiber length, resulting in a better seepage effect. If the fiber is too long, it will be easily twisted in the soil mass, thereby elongating the seepage passage and even blocking the seepage passage and then reducing the soil permeability, which is consistent with the conclusion in Reference [31]. Figure 4b is the time–history curve of the disintegration rate of loess reinforced by basalt fibers of different lengths. As shown in the figure, the final disintegration time of reinforced soil also gradually increases by increasing the fiber length. If the fiber content is 1.00% and the fiber length is 4 mm, 8 mm, and 12 mm, the disintegration time of the reinforced soil is 970 s, 1030 s, and 1170 s longer than that of plain soil. By increasing the fiber length, the friction and cohesive force between fiber and soil become stronger, and the scope of action becomes wider, thus delaying soil cracking in water.

**Figure 5:**
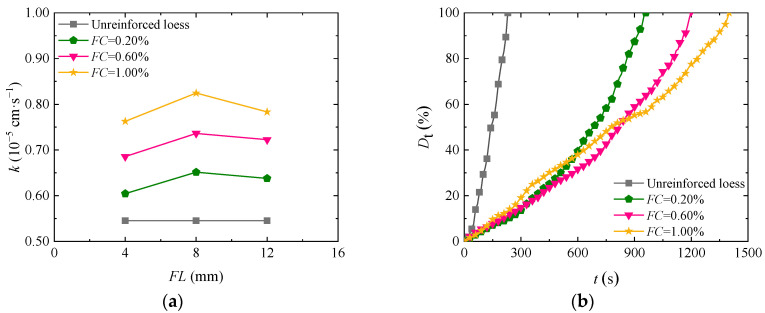
Impact of basalt fiber content on loess permeability coefficients and disintegration rate: (**a**) permeability characteristics; (**b**) disintegrating characteristics.

To analyze the impact of basalt fiber content on loess permeability and disintegration characteristics, Figure 5a presents the curve of permeability coefficient change with fiber lengths in the loess reinforced by basalt fiber with different fiber content under a confining pressure of 100 kPa. As shown in the figure, if the basalt fiber content is constant, the permeability coefficient of loess increases first and then decreases as the fiber length increases. If the fiber length is constant, the permeability coefficient of fiber-fortified soil rises by increasing the fiber content. The main reason is that adding basalt fiber increases the seepage passage in the soil mass. By increasing the fiber content, the seepage passages due to fibers in the soil mass also increase, thus improving the permeability coefficient of the soil mass. This finding is consistent with the conclusion in Reference [32]. Figure 5b presents the time–history curve of the disintegration rate of loess fortified by basalt fibers of different content. As shown in the figure, the final disintegration time of fiber-fortified soil shows a gradually increasing trend as the fiber content increases. The main reason for the above situation is that when the fiber length is constant, the effect of twisted and interwoven fibers is more significant in the soil, and the bridging effect between fibers is also more significant, and these effects have delayed the damage to the soil structure, thereby increasing the disintegration time.

### 3.3. Analysis of Permeability and Disintegration Characteristics of the Loess Solidified by Guar Gum and Basalt Fiber

**Figure 6:**
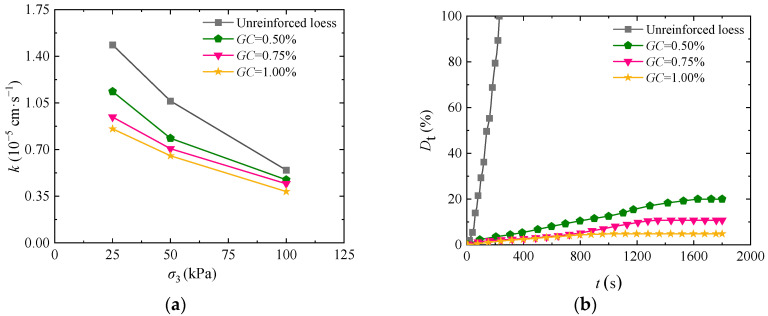
Impact of guar gum content on the permeability coefficients and disintegration rate of solidified loess: (**a**) *FL* = 8 mm, *FC* = 1.00%; (**b**) *FL* = 8 mm, *FC* = 1.00%.

To study the impact of guar gum content on the permeability and disintegration characteristics of the loess solidified by guar gum and basalt fiber, Figure 6a presents the curve of permeability coefficient change with guar gum content in the solidified loess under different confining pressures with an 8 mm fiber length and a fiber content of 1.00%. Figure 6b shows the time–history curve of the disintegration rate of solidified loess under different guar gum contents when the fiber length is 8 mm and the fiber content is 1.00%. As shown in the figure, the permeability coefficient and disintegration rate of the loess jointly solidified by guar gum and basalt fiber show a gradually decreasing tendency to decrease with increasing guar gum content when the fiber content and fiber length are constant. When the guar gum content is 0.50%, 0.75%, and 1.00%, the permeability coefficient and disintegration rate of solidified loess are decreased by 10.40%, 12.90%, and 13.50%, respectively, and 4.80%, 10.70%, and 19.90%, respectively. The water stability of cement combined with fiber-solidified loess is improved in the range of 40–80% [14], and that of guar gum combined with basalt fiber-solidified loess is improved in the range of 60–80%. The main reason for this situation is that after guar gum is added to the soil mass, it produces gel In contact with water and the gel makes the particles in the soil bond more closely and fill the pores in the soil mass so that pore passages in the soil mass are blocked and the process of water erosion is delayed for the sample. The combined effect of guar gum and basalt fiber makes soil particles bond more closely and blocks seepage passages to reduce soil permeability and disintegration.

**Figure 7:**
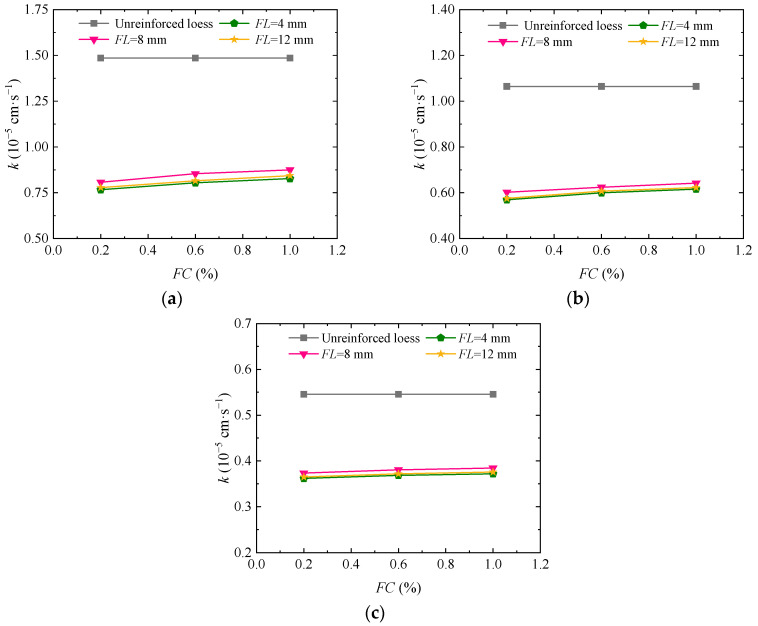
Impact of basalt fiber length on the permeability coefficient of the loess jointly solidified by guar gum and basalt fiber: (**a**) *σ*_3_ = 25 kPa; (**b**) *σ*_3_ = 50 kPa; (**c**) *σ*_3_ = 100 kPa.

Figure 7a–c shows the curve of permeability coefficient change with fiber length when the guar gum content in the loess, jointly fortified by guar gum and basalt fiber, is 1.00%. As shown in the figure, the permeability coefficient of the loess jointly solidified by guar gum and basalt fibers becomes larger and then smaller with increasing fiber lengths when the confining pressure and guar gum content are constant. The growth rate of the loess permeability coefficient in the fiber content range of 0.20% to 0.60% is significantly greater than in the fiber content range of 0.60% to 1.00%. When the confining pressure is 25 kPa, 50 kPa, and 100 kPa, and the fiber content is 0.20%, 0.60%, and 1.00%, the permeability coefficient of solidified loess is 26.90% to 31.90% lower than that of reinvented soil. As for the primary cause of this situation, the length of seepage passages in the soil jointly solidified by guar gum and basalt fiber increases by increasing the fiber length at constant fiber content, so the soil permeability is improved. If the fiber is too long, it is easily twisted, which will reduce the permeability of solidified loess.

**Figure 8:**
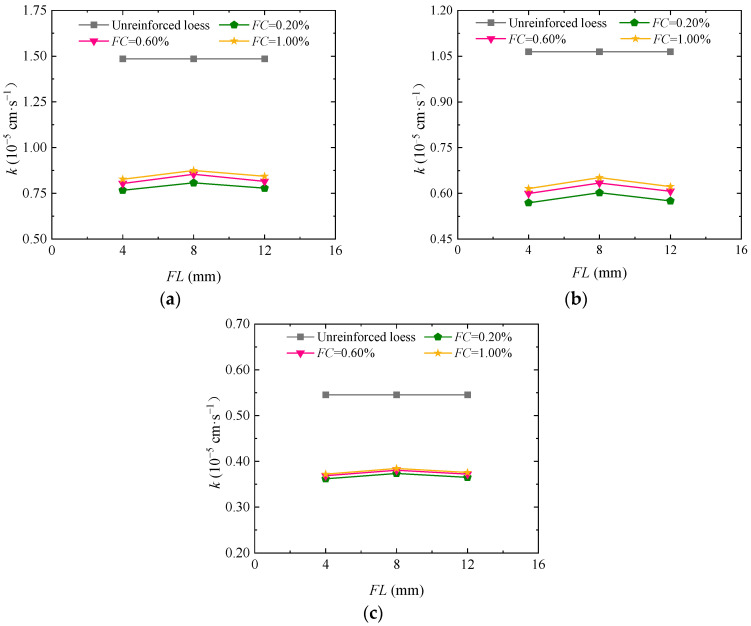
Impact of basalt fiber content on the permeability coefficient of the loess jointly solidified by guar gum and basalt fiber: (**a**) *σ*_3_ = 25 kPa; (**b**) *σ*_3_ = 50 kPa; (**c**) *σ*_3_ = 100 kPa.

Figure 8a–c shows the curve of permeability coefficient change with fiber content when the guar gum content in the loess jointly fortified by guar gum and basalt fiber is 1.00%. As shown in the figure, the permeability coefficient of the loess jointly solidified by guar gum and basalt fiber shows a tendency to slow with increasing fiber content when the confining pressure and guar gum content are constant. When the confining pressure is 25 kPa, 50 kPa, and 100 kPa, the fiber content is 0.20%, 0.60%, and 1.00%, and the fiber length is 4 mm, 8 mm, and 12 mm, the permeability coefficient of solidified loess decreases by 30.50% to 31.90%. This is because guar gum has the effect of reducing loess permeability; the random distribution of fibers in the soil mass increases the internal seepage passages to improve the permeability of solidified loess. By increasing the fiber content, there are more and more seepage passages generated by the fiber in the soil mass, so the soil permeability increases gradually.

### 3.4. Analysis of the Mechanism of Action for the Loess Solidified by Guar Gum and Basalt Fiber

**Figure 9:**
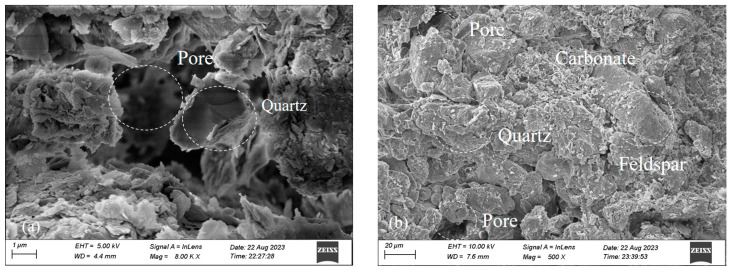

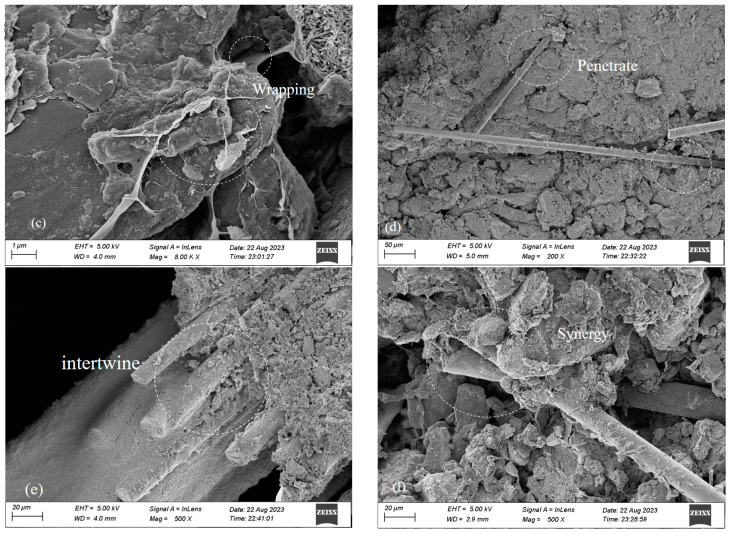
The microscopic structure of the loess jointly solidified by guar gum and basalt fiber: (**a**) microstructure; (**b**) mineral composition; (**c**) wrapping effect; (**d**) penetrate effect; (**e**) intertwine effect; (**f**) glue-bar synergy effect.

As shown in Figure 9a,b, loess has large pores, multiple mineral compositions, and a loose arrangement of soil particles, resulting in high permeability of loess [33]. To study the mechanism of action for the loess jointly solidified by guar gum and basalt fiber, Figure 9c presents the guar gum that reacts with water to generate hydrogel. With strong fluidity, the hydrogel can effectively fill pores, make the soil sample compact, and block water penetration to improve the soil’s strength. Figure 9d shows that basalt fibers penetrate the soil to form a seepage channel. As the fiber content increases, the soil will generate multiple seepage channels, thereby improving the permeability of the loess. Figure 9e shows that when the fibers are too long, they become intertwined, reducing the permeability of the solidified soil. Figure 9f presents the interaction of guar gum and basalt fiber in solidified loess; the basalt fiber is evenly distributed in the soil mass, and the relative slip of soil particles is restricted by the twisting, embedding, and bridging effects of every single fiber. A mesh support can be formed by multiple fibers, and the mesh structure restricts the deformation and displacement of soil particles. The movement of soil particles is greatly restricted in this way. After hydration, guar gum tightly locks the basalt fiber to form an anchorage zone. The hydrate of guar gum fills part of the pores in the soil sample and improves the soil compactness to increase the contact surface between fiber and soil medium and the interaction force between interfaces and provide the fiber-fortified soil with better mechanical properties. This is consistent with the conclusion in Reference [28].

### 3.5. XDR Diffraction Analysis Test for the Loess Solidified by Guar Gum and Basalt Fiber

**Figure 10:**
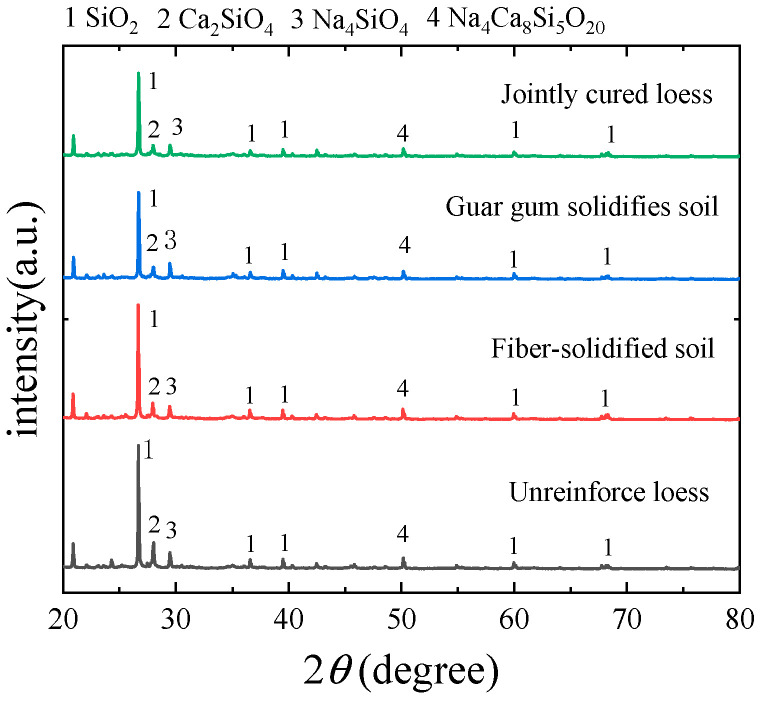
XRD diffraction analysis.

XRD diffraction analysis tests were carried out on the specimens of the untreated loess, guar gum-solidified loess, basalt fiber-reinforced loess, and combined guar gum and basalt fiber-solidified loess, and the results obtained are shown in Figure 10. As can be seen from the figure, the main components in each specimen are dominated by SiO_2_, Ca_2_SiO_4_, Na_4_SiO_4_, and Na_4_Ca_8_Si_5_O_20_. Comparing the positions of the peaks in the graphs, it can be found that the basalt fibers and guar gum do not change the morphology of the soil, and the SiO_2_ and other small amounts of substances with higher peaks do not change the morphology of the soil by mixing. The higher peaks of SiO_2_ and other small amounts of substances do not have obvious changes due to the mixing of basalt fibers and guar gum, which indicates that the microstructure changes in the soil are not caused by the production of new substances.

## 4. Modeling of Permeability of Loess Jointly Solidified by Guar Gum and Basalt Fiber

### 4.1. Model Building

In the permeability process of cured soil, the viscous substance formed by guar gum when it meets water is mainly a water-soluble polysaccharide gum. This polysaccharide gum is formed by the expansion of guar gum molecules in water and has strong viscosity properties, which reduces permeability, while the length and dosage of basalt fibers will form a seepage channel in the soil so that the permeability of the soil body becomes larger. With the increasing perimeter pressure, the soil body is gradually compacted, the voids in the soil are reduced, and the permeability decreases. In the case of joint reinforcement of the soil body by glue-tendon, guar gum will adhere to the surface of the fiber, and the joint effect of glue-tendon stabilizes the movement between particles in the soil and plays some effect of compression resistance, so the peripheral pressure increases, the permeability of the soil body will still be reduced, but the magnitude of the decline is small.

Building on the above study and considering the dimension impact, it is assumed that the permeability coefficient k of the fortified soil is a function of *FL*, *FC*, *GC*, and *σ*_3_.

According to the geotechnical testing standard (GB/T50123-2019) [29], the test confining pressure was determined to be 25 kPa, 50 kPa, and 100 kPa by sampling depth.

The longer the fiber length, the easier it is to be entangled and knotted in the soil, which affects the soil permeability.

The *FL* is less than or equal to 8 mm.

(2) $$\frac{k_{GFR}}{k_{0}}=f\left(\frac{FL+0.5FC}{0.16n\sigma_{3}\cdot GC+FL}\right)$$

where *k_GRF_* is the coefficient of permeability of cured loess, *k*_0_ is the coefficient of permeability of remodeled soil at 25 kPa perimeter pressure, *FL* is the fiber length, *FC* is the fiber content, *GC* is the guar gum content, *σ*_3_ is the perimeter pressure, and n is 0.625 times *FL*/4.

The *FL* is greater than 8 mm.

(3) $$\frac{k_{GFR}}{k_{0}}=f\left(\frac{FL+FC}{0.16n\sigma_{3}\cdot GC+1.2FL}\right)$$

By plotting this function curve through the test results, it is found that there is a linear relationship.

The *FL* is less than or equal to 8 mm, which is given by Equation (2).

(4) $$k_{GFR}=a+b\left(\frac{FL+0.5FC}{0.16n\sigma_{3}\cdot GC+FL}\right)k_{0}$$

The *FL* is greater than 8 mm, which is given by Equation (3).

(5) $$k_{GFR}=a+b\left(\frac{FL+FC}{0.16n\sigma_{3}\cdot GC+1.2FL}\right)k_{0}$$

where the parameters *a* and *b* are the intercept and slope of the fitted curve.

### 4.2. Model Parameter Fitting

**Figure 11:**
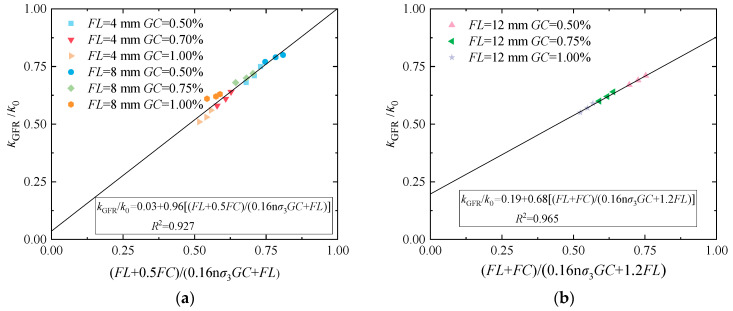
Fitted curve of permeability coefficient. (**a**) *FL* = 4 mm and *FL* = 8 mm; (**b**) *FL* = 12 mm.

Figure 11a shows the fitted effect of cured loess with 0.50%, 0.75%, and 1.00% guar gum content; 0.20%, 0.60%, and 1.00% fiber content; and 4 mm and 8 mm fiber length at 25 kPa confining pressure. Figure 11b shows the fitted effect of cured loess with 0.50%, 0.75%, and 1.00% guar gum content; 0.20%, 0.60%, and 1.00% fiber content; and 12 mm fiber length at 25 kPa confining pressure. It is possible to see that the fitting impact is fundamentally positive and correlated with a good linear relationship. From Figure 11, it is possible to see that the permeability coefficient of cured loess is negatively correlated. The content of guar gum is positively correlated with basalt fiber length and content. Through the regression analysis, a and b parameter values can be obtained: if the fiber length is less than or equal to 8 mm, the curve of the fit of the decidability coefficient is 0.927; if the fiber length is greater than 8 mm, the curve of the fit of the decidability coefficient is 0.965. This indicates that there is a good linear relationship between the horizontal and vertical axes of the model, which can be represented by a linear equation. When the fiber length is 4 mm and 8 mm, a is 0.96, b is 0.03, standard error a is 0.06531, and standard error b is 0.0426. When the fiber length is 12 mm, a is 0.68, b is 0.19, standard error a is 0.017, and standard error b is 0.01078.

### 4.3. Model Verification

**Figure 12:**
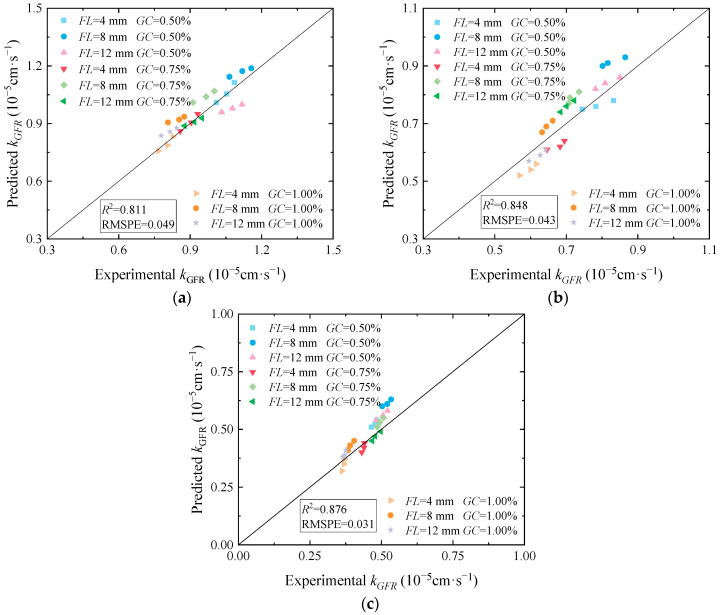
Comparison of permeability coefficients between experimental and predicted values: (**a**) *σ*_3_ = 25 kPa; (**b**) *σ*_3_ = 50 kPa; (**c**) *σ*_3_ = 100 kPa.

To verify the model’s reliability, the parameters a and b were applied to Equations (4) and (5) for fiber lengths less than or equal to 8 mm and greater than 8 mm, and the test data of the permeability coefficient of the cured soil under the confining pressures of 25 kPa, 50 kPa, and 100 kPa were substituted into the calculation. The permeability coefficient of cured soil can be obtained. As shown in Figure 12a–c, the predicted data of the permeability coefficient of cured loess compared with the test data indicate that the permeability coefficient is more uniformly distributed on both sides of the parallel lines. It shows that the predicted permeability coefficient results are more in line with the test results, indicating that the model is suitable for predicting the permeability coefficient of loess jointly solidified by guar gum and basalt fiber.

## 5. Conclusions

By conducting permeability and disintegration tests and studying the water sensitivity of the loess jointly solidified by guar gum and basalt fiber, analyzing the impact of guar gum content, fiber length, fiber content, and other related factors on the permeability and disintegration characteristics of the solidified loess, and obtaining the optimal solidification conditions, the microscopic structure of the loess is constructed based on the scanning electron microscopy (SEM) test, unveiling the synergistic solidification mechanism of guar gum and basalt fiber. On this basis, the permeability model of combined glue-reinforcement-fortified loess is established and verified. The main conclusions are as follows:

(1) The permeability of the loess strengthened through guar gum decreases as the confinement pressure increases. Under the same confining pressure, the permeability of solidified loess gradually decreases with increasing guar gum content. Compared with untreated soil, the permeability coefficient of guar gum-solidified soil was reduced by 50.50% under a confining pressure of 25 kPa. The disintegration rate of guar gum-solidified loess decreased significantly with the increase in guar gum content. When the guar gum content is 1.00%, the disintegration rate of solidified soil is reduced by 94.1%. After a specific period, the sample disintegration rate steadily tends to be stable. By combining the permeability characteristics with the disintegration characteristics of solidified loess, we can learn that the optimal guar gum content is 1.00%.
(2) If the fiber content is constant, the permeability of the basalt fiber-fortified loess increases first, followed by a decreasing trend as the fiber length increases. Compared with untreated soil, when the fiber length is 8 mm, the permeability coefficient of solidified loess is increased by 12.70%. With the increase in fiber content, the permeability of fiber-reinforced soil increased gradually. The final disintegration time of fiber-fortified soil shows a little-by-little increase with the increase in fiber content and fiber length. For constant fiber content and length, the permeability and disintegration of the loess jointly solidified by guar gum and basalt fiber show a gradually decreasing trend with the increase in guar gum content. The results showed that the optimal solidification conditions were as follows: guar gum 1.00%, basalt fiber length 12 mm, and content 1.00%. At this time, the permeability coefficient and disintegration rate of solidified soil were reduced by 31.90% and 95.2%, respectively.
(3) Guar gum reacts with water to generate hydrogel, which has strong fluidity and can effectively fill pores. However, overdosing on guar gum will lead to a significant decrease in soil permeability, which is not conducive to vegetation growth. Basalt fibers are distributed evenly in the soil mass, and mesh support can be formed by multiple fibers to restrict the deformation and displacement of soil particles. However, an excess of fibers will lead to the separation of soil particles, which will destroy the soil structure. The fiber in the loess, jointly solidified by guar gum and basalt fiber, can increase the adhesion area of the cementing agent and better promote the cementing effect. Compared with the composite of cement and fiber-reinforced loess, the combination of guar gum and basalt fiber-reinforced loess has better water stability.
(4) The experimental results are in good agreement with the predicted experimental results, indicating that the established model and parameter estimation method are suitable for predicting the permeability coefficient of guar gum basalt fiber combined with reinforcement of loess.
(5) The use of guar gum combined with basalt fiber to strengthen loess can improve the stability of the slope and strengthen the roadbed. The research results of this paper can provide a reference for soil solidification engineering.
(6) In practical engineering, the addition of basalt fiber and guar gum content should consider construction conditions and costs. In the future study, dry–wet cycle tests and freeze–thaw cycle tests will be conducted to study the durability of solidified soil under different environments.

## Figures and Tables

**Table 1:** The basic physical properties of Loess.

Water Content (%)	Dry Density (g·cm^−3^)	Specific Gravity Gs	Initial Porosity Ratio	Plasticity Limit (%)	Liquid Limit (%)	Plasticity Index
14.50	1.24	2.67	0.73	18.60	33.80	15.20

**Table 2:** Physical and mechanical indicators of basalt fiber.

Density (g·cm^−3^)	Diameter (μm)	Tensile Strength (MPa)	Elastic Modulus (GPa)	Elongation at Break (%)	Acid and Alkali Resistance
2.65	10	3500~4500	100	2.20	Strong

**Table 3:** Triaxial permeability test scheme.

Confining Pressure *σ*_3_ (kPa)	Fiber Length *FL* (mm)	Fiber Content *FC* (%)	Guar Gum Dosage *GC* (%)
25	0	0	0, 0.50, 0.75, 1.00
	4	0.20, 0.60, 1.00	0, 0.50, 0.75, 1.00
	8	0.20, 0.60, 1.00	0, 0.50, 0.75, 1.00
	12	0.20, 0.60, 1.00	0, 0.50, 0.75, 1.00
50	0	0	0, 0.50, 0.75, 1.00
	4	0.20, 0.60, 1.00	0, 0.50, 0.75, 1.00
	8	0.20, 0.60, 1.00	0, 0.50, 0.75, 1.00
	12	0.20, 0.60, 1.00	0, 0.50, 0.75, 1.00
100	0	0	0, 0.50, 0.75, 1.00
	4	0.20, 0.60, 1.00	0, 0.50, 0.75, 1.00
	8	0.20, 0.60, 1.00	0, 0.50, 0.75, 1.00
	12	0.20, 0.60, 1.00	0, 0.50, 0.75, 1.00

**Table 4:** Disintegration test scheme.

Fiber Length *FL* (%)	Fiber Content *FC* (%)	Guar Gum Dosage *GC* (%)
0	0	0, 0.50, 0.75, 1.00
4	0.20, 0.60, 1.00	0, 0.50, 0.75, 1.00
8	0.20, 0.60, 1.00	0, 0.50, 0.75, 1.00
12	0.20, 0.60, 1.00	0, 0.50, 0.75, 1.00
0	0	0, 0.50, 0.75, 1.00
4	0.20, 0.60, 1.00	0, 0.50, 0.75, 1.00
8	0.20, 0.60, 1.00	0, 0.50, 0.75, 1.00
12	0.20, 0.60, 1.00	0, 0.50, 0.75, 1.00
0	0	0, 0.50, 0.75, 1.00
4	0.20, 0.60, 1.00	0, 0.50, 0.75, 1.00
8	0.20, 0.60, 1.00	0, 0.50, 0.75, 1.00
12	0.20, 0.60, 1.00	0, 0.50, 0.75, 1.00

## Data Availability

All data generated or analyzed during this study are included in this published article.
